# Decoding population PM_2.5_ exposure in China: interplay of emissions, meteorology, and inequality (2013–2020)

**DOI:** 10.3389/fpubh.2025.1577897

**Published:** 2025-08-11

**Authors:** Sujing Li, Chenxi Wang, Linmeng Ma, Xingxing Wang, Guolei Du, Changhao Wu

**Affiliations:** ^1^State Key Laboratory of Vegetation Structure, Function and Construction (VegLab), Yunnan University, Kunming, China; ^2^Yunnan International Joint Laboratory of Monsoon and Extreme Climate Disasters, Yunnan University, Kunming, China; ^3^Yunnan Key Laboratory of Eteorological Disasters and Climate Resources in the Greater Mekong Subregion, Yunnan University, Kunming, China; ^4^The Institute of China’s Science, Technology and Education Policy, Zhejiang University, Hangzhou, China; ^5^School of Economics and Management, Guizhou Normal University, Guiyang, China; ^6^Business School, Shandong Jianzhu University, Jinan, China; ^7^Guangzhou Huashang College, Guangzhou, China; ^8^Shandong University of Finance and Economics, Jinan, China; ^9^School of Geography, Earth and Environmental Sciences, University of Birmingham, Birmingham, United Kingdom

**Keywords:** environmental inequality, PM_2.5_ exposure, meteorological normalization, emission burden, environmental governance

## Abstract

Over the past decade, China has significantly improved air quality by integrating environmental policies with economic growth. Yet, environmental inequality remains a major challenge to social equity and sustainable development. This study examines the socioeconomic impacts of PM_2.5_ exposure using population data from 1,317 county towns across 32 provinces (2013–2020), employing meteorological normalization and population-weighted exposure indices. The findings reveal that lower-income regions (L4) achieved the highest PM_2.5_ reduction (54%), whereas wealthier regions (L1–L3), despite higher pollution levels, saw lower reductions (45–50%), highlighting an unequal emission reduction burden. PM_dw exhibits more stable spatiotemporal patterns than PM_2.5_, offering clearer insights into emission trends. Despite overall improvements, residents in less-developed areas still face higher exposure, while urban centers, benefiting from more resources, experience increased health risks. Vulnerable populations—including coal miners, the educated, women, and the older adult—disproportionately suffer from high exposure levels. Meteorological conditions have generally mitigated PM_2.5_ exposure, with the most significant dispersion effect in 2018. Notably, meteorology’s role in mitigating inequality in occupational exposure significantly decreased from 43.7% in 2013 to 4.5% in 2019, while its exacerbating effect on urban–rural inequality, contributing 43.7% in 2010, drastically reduced by 2020, even shifting to a slight alleviating role. To achieve equitable environmental governance and robust pollution control, policies must not only address regional economic disparities and prioritize protection for disadvantaged communities but also account for the complex and evolving modulating role of meteorological conditions on exposure inequality.

## 1 Introduction

Since the turn of the century, China has been at the forefront of global development, with its coal consumption peaking at 91.94 exajoules in 2023, making it one of the world’s largest consumers (1). This significant energy use contributes to substantial emissions of particulate matter (PM_2.5_), closely linked to adverse health outcomes (2–5). Research consistently highlights the detrimental effects of PM_2.5_ on health, significantly increasing risks of respiratory diseases and reducing life expectancy (6–11). In response, the Chinese government has implemented robust policies like the “Ten Rules of the Atmosphere” and the “Battle of the Blue Sky,” achieving a 54% reduction in PM_2.5_ levels and maintaining good air quality on over 86% of days annually in key cities (12). Despite these improvements, disparities in air quality exposure continue to pose challenges, highlighting persistent environmental inequalities.

Recent research highlights the persistent environmental inequalities associated with air pollution, which manifest not only in pollution distribution but also in disparate access to environmental protection and resources across social strata. This disparity is most evident in areas benefiting from natural advantages or suffering from industrial pollution. Factors such as geography, industrial density, and economic development play significant roles in the distribution of these inequalities (13–16). Sociological analyses suggest that rural migrants and lower economic sectors face heightened risks due to their proximity to pollution-intensive industries (17–21). Furthermore, demographic shifts towards an aging population are exacerbating the health impacts of PM_2.5_, significantly increasing mortality rates, particularly among women aged 30–45, who experience double the pollutant exposure of the general adult population (22–26). These conditions undermine the benefits of air quality improvements and healthcare advancements, ultimately impacting subjective well-being and mental health.

Despite numerous studies addressing the environmental inequalities caused by air pollution in China, there remains a significant gap in comprehensive analysis of pollution exposure disparities across different socio-economic groups, particularly in terms of the specific roles of meteorological and emission factors. This study addresses this gap by systematically assessing the PM_2.5_ exposure conditions in 1,317 county towns across 32 provinces in mainland China from 2013 to 2020. Utilizing a meteorological normalization model built through the integration of spatial information and machine learning techniques (27, 28), combined with a population-weighted pollution exposure index (29), we meticulously analyzed exposure disparities across various socio-economic dimensions including urban–rural settings, occupation, age, gender, and educational levels, as well as the impacts of meteorological factors. Through exhaustive data analysis, this paper reveals regional variations and the effectiveness of China’s policies for reducing atmospheric emissions, highlighting the socio-economic impacts of these policies, and discussing environmental health inequalities from a community perspective. Our findings aim to provide a scientific basis for the formulation of more equitable environmental policies and advocate for enhanced health rights for socio-economically disadvantaged groups.

## 2 Method

### 2.1 Data sources

This study utilizes data from multiple sources, including the National Bureau of Statistics, the 6th and 7th Population Censuses, the European Centre for Medium-Range Weather Forecasts (ECMWF), and the China National Environmental Monitoring Centre (CNEMC). Specific information on each data source is shown in Table 1. The National Bureau of Statistics provides annual county-level population data for sectors such as mining, education, manufacturing, and transportation from 2013 to 2020, along with county-level education qualification data. The 6th and 7th Population Censuses offer data on sex, age groups, and place of residence (urban, rural, town) for 2010 and 2020, collected every 10 years. The CNEMC platform provides national PM_2.5_ site-level hourly data from 2013 to 2020 (30), aggregated into annual averages for analysis. To ensure consistency, census data from different years were standardized. Data for 2005 and 2015 were obtained from 1% population sample surveys, while data for other years were sourced from 1‰ population change sample surveys. To account for these varying sampling ratios and ensure comparability, we inversely estimated the total population based on their respective sampling proportions. Specific details on the sampling ratios are available in the “China Statistical Yearbook” publications in National Bureau of Statistics^1^.

**Table 1:** Meteorological, air quality, and population data sources (2013–2020).

Name	Unit	Data type	Data sources	Time span
Mean sea level pressure	Pa	Hourly/0.25°	ECMWF	2013–2020
Relative humidity	%			
2 m temperature	K			
10 m u-component of wind	m/s			
10 m v-component of wind	m/s			
Boundary layer height	m			
Population occupational data	Million	Annual, County-level	National Bureau of Statistics	2013–2020
Population gender and age data	Sort			
Population education	Million	Every ten years, County-level	Sixth and Seventh National Population Census	2010,2020
Residence and household registration	Million			
PM_2.5_ measured data	ug/m^3^	Hourly/Station	CNEMC	2013–2020

Meteorological data from the ECMWF, with a spatial resolution of 0.25° × 0.25° from 2013 to 2020, were used as covariates for PM_2.5_ inversion (31). While this resolution may not fully capture highly localized meteorological variations, such as those in complex urban microclimates or specific complex terrains, it provides the most comprehensive and consistently available long-term reanalysis data for nationwide studies like ours, ensuring broad spatial and temporal coverage for our analysis across 1,317 county towns. The data include mean sea level pressure (msl, Pa), relative humidity (rh, %), temperature (t2m, K), easterly and northward wind components (10 U and 10 V, m/s), and boundary layer height (blh, m). These data were localized based on the latitude and longitude of each PM_2.5_ station, enabling the extraction of meteorological parameters for each site.

Our study implemented a stringent quality control and preprocessing protocol for daily PM_2.5_ data to ensure analytical reliability. Physically invalid PM_2.5_ concentrations (values less than 0 μg/m^3^ or greater than 1,000 μg/m^3^) were directly excluded. For the remaining valid data, outliers were identified using a ± 3 standard deviation rule within a 15-day sliding window, complemented by a review of global 0.1 and 99.9% percentiles to confirm extreme values. Identified outliers were replaced via linear interpolation to maintain time series continuity. PM_2.5_ Missing data were primarily imputed using linear interpolation, with a maximum imputation window of 15 continuous days; longer gaps were left unfilled to prevent the introduction of highly uncertain synthetic data. Importantly, the meteorological parameters obtained from ECMWF’s ERA5 reanalysis product inherently provide complete spatiotemporal coverage and therefore did not require any missing value imputation in our study. This rigorous data processing supports the reliability of our machine learning algorithms (57).

### 2.2 Estimates of PM_2.5_ population exposure

Due to the uneven spatial distribution of PM_2.5_ monitoring stations, even at the county level, we need to apply kriging interpolation to seamlessly fill gaps in station PM_2.5_ data and subsequent DW data. The processed seamless data is then spatially matched with population data to more accurately estimate the population-weighted PM_2.5_ exposure concentration.

Due to the uneven spatial distribution of PM_2.5_ monitoring stations across China, Kriging interpolation was applied to both raw PM_2.5_ and deweathered PM_2.5_ (PM_dw) station data to generate spatially continuous maps, ensuring comprehensive coverage for subsequent analyses. The deweathering (DW) method was used to separate meteorological influences from emission signals, deriving PM_dw. Using the spatially continuous PM_dw after Kriging interpolation for population exposure calculations helps prevent misjudgments of concentration errors caused by uneven station distribution, while also decoding the extent to which meteorological factors influence population exposure. Finally, the processed seamless data were spatially matched with population data to more accurately estimate population-weighted PM_2.5_ exposure.

Population-Weighted PM_2.5_ Exposure Estimation (32) was used in this study: Suppose there are i county towns (which serve as our basic analytical units across the nation), with the PM_2.5_ concentration of the i_th_ province denoted as $PM_{2.5,i}$ , and its population as $P_{i}$ . The calculation Equation 1 for the population-weighted PM_2.5_ exposure concentration for different groups is as follows Equation 1:


(1) $${\bar{\mathrm{PM}}}_{2.5}=\frac{\sum_{i=1}^{n}\;\;(PM_{2.5,i}\times P_{i})}{\sum_{i=1}^{n}\;\;P_{i}}\;$$

Where:

${\bar{\mathrm{PM}}}_{2.5}$ represents the population-weighted average PM_2.5_ concentration.

$PM_{2.5,i}$ is the PM_2.5_ concentration of the i_th_ county town.

$\;P_{i}$ is the population of the ith county town.

n is the total number of county town (e.g., 1,317 for the national average).

### 2.3 Spatial information embedded random forest

Given that the study area spans the entire country, the characteristics of PM_2.5_ vary significantly across different regions. Traditional deweathering methods using Random Forest Pointwise Models (RF Pointwise Model) suffer from low prediction accuracy due to limited sample sizes. Meanwhile, Random Forest Holistic Models (RF Holistic Model) tend to overwhelm the features of regions with fewer samples because of uneven sample distribution. To address these issues, this study employs a Geographically Weighted Random Forest (GWRF) model for simulating and predicting PM_2.5_. GWRF is a spatial analysis method that integrates spatial weight matrices with the Random Forest model, designed to handle datasets with significant spatial heterogeneity (17). GWRF assigns different weights to different samples during the decision tree construction process by incorporating the influence of geographic location, thereby improving predictive accuracy. This method can capture local patterns in spatial data, which is particularly suitable for data with uneven spatial distribution. The mathematical expression of the GWRF model is as follows Equation 2:


(2) $$Y_{i}=\beta_{0}(u_{i},v_{i})+\sum_{k=1}^{p}\;\beta_{k}(u_{i},v_{i})X_{\mathrm{ik}}+\varepsilon_{i}\;$$

Where $Y_{i}$ represents the dependent variable at location $i,\beta_{0}$ is the intercept term, $\left(u_{i},v_{i}\right)$ are the coordinates of location $i,\beta_{k}$ is the regression coefficient of the k -th independent variable, and $\varepsilon_{i}$ is the error term. In this way, the GWRF model provides a customized predictive model for each geographic location, better reflecting the local characteristics of spatial data. By incorporating geographical weights, the GWRF model ensures sufficient data coverage while preserving the features of regions with smaller sample sizes. These geographical weights are crucial for estimating the local regression coefficients $\beta_{k}(u_{i},v_{i})$ at each location i. Specifically, they are determined by a distance-decay function, such as the Gaussian kernel function. This function assigns higher weights to observations closer to location i and lower weights to those further away. The extent of this spatial influence is controlled by a crucial parameter called the bandwidth, which defines the decay rate of the weights with increasing distance. In our study, the optimal bandwidth was determined using a cross-validation (CV) approach, minimizing the prediction error. In this study, we trained and compared three model architectures of the Random Forest: GWRF, RF Holistic Model, and RF Pointwise Model, to examine the impact of geographical weighting. The final model aggregates predictions from multiple geographically weighted trees, offering a robust and spatially adaptive framework for urban PM_2.5_ date prediction.

### 2.4 Deweather method

**Figure 1:**
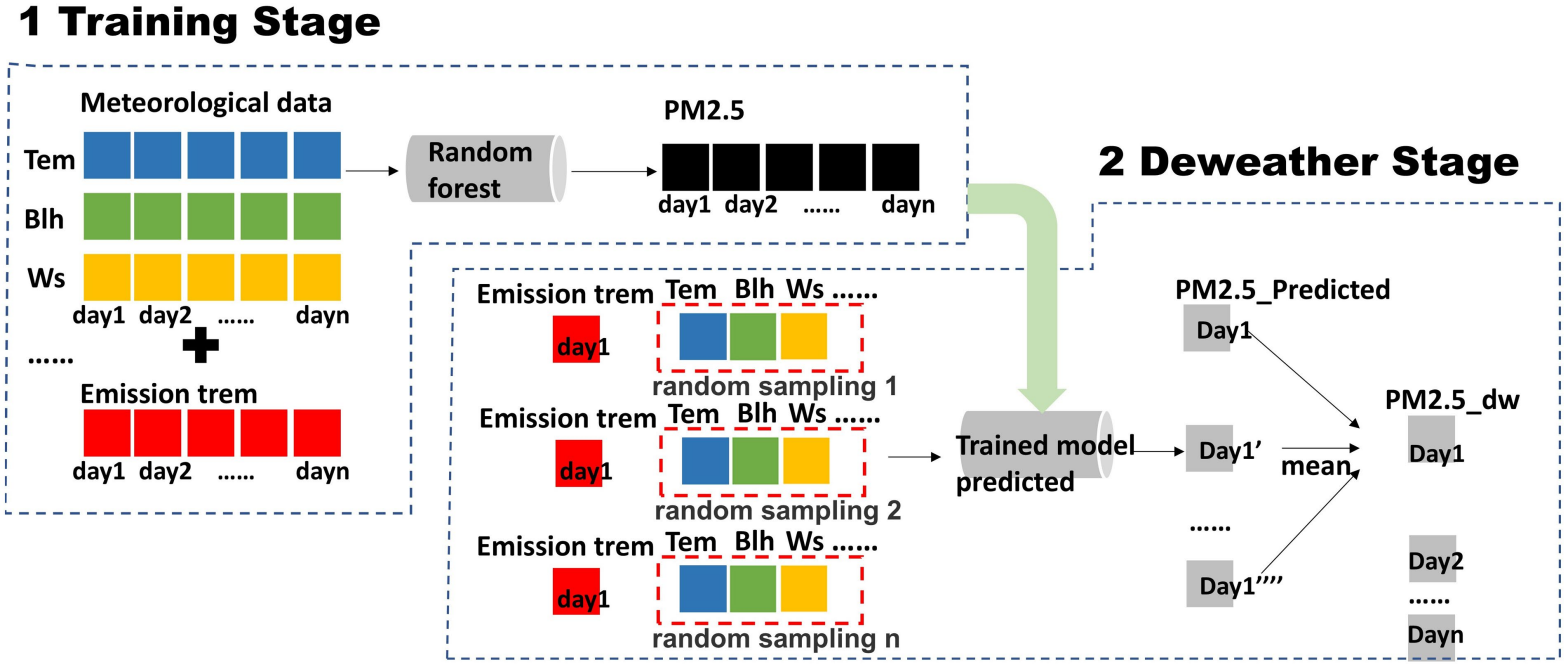
Basic principle diagram of deweather.

The effect of meteorological fluctuations can be removed by the Deweather method to obtain the PM_2.5_ concentration under average meteorological conditions termed PM_dw (33), and the principle of Deweather operation is shown in Figure 1, and the configuration operation is as follows:

Parameter settings of Deweather: The output parameter PM_2.5_ (ug/m^3^), and the meteorological part of the input parameters include: Mean sea level pressure (msl, Pa), relative humidity (rh, %), temperature (t2m, K), eastward component of 10 m wind (10 U, m/s), northward component of 10 m wind (10 V, m/s), and boundary layer height (blh, m). Time terms representing emissions include: Normalized seasonal coefficient (NSC) (34), Day of Week, Unix time of the observation (number of seconds since 1 January 1970). Our selection of the current meteorological parameter set is based on a careful consideration of our core research objective and data processing strategy. We aim to more effectively identify and separate long-term, stable emission-driven signals (PM_dw) through the deweathering method, thereby revealing emission trends and the actual effects of environmental policies. While instantaneous and highly variable meteorological conditions (such as direct precipitation intensity or high-frequency vertical wind speeds) have physical impacts on PM_2.5_ concentrations, they can introduce high-frequency noise in the short term, potentially interfering with the identification of smoother, more representative emission trends. Therefore, we prioritized parameters that can capture the characteristics of the average meteorological field and contribute to a stable PM_dw curve. The construction of the model’s inputs and outputs is shown in Equation 3.


(3) $$\begin{array}{l} {\mathrm{PM}}_{2.5}=f(\mathrm{msl},\mathrm{rh},t2m,10U,10\;V,\mathrm{blh},\mathrm{NSC},\\ \;\;\;\;\;\;\;\;\;\;\;\;\;\;\;\;\;\;\;\;\;\;\;\;\;\mathrm{Day}\;\text{of Week},\text{Unix Time}) \end{array}$$

Base model hyperparameter setting: construct a PM_2.5_ estimation model based on the GWRF algorithm, the number of trees is set to 500, the training set is 70%, the validation set is 10%, and the test set is 20%, and the 5-fold cross-validation is used to avoid obtaining overfitting error results. Finally, a random forest based PM_2.5_ estimation model is trained.

Deweather method: The time terms (NSC, Day of Week, Unix time) are fixed for all data samples, and the meteorological part uses multiple resamples to construct multiple randomly selected meteorological conditions for each data sample, and the database of the extracted meteorological fields is drawn from the complete meteorological data of all time scales of all stations in the country. The purpose is to ensure that the Deweather results for all data are obtained in the same meteorological field context and thus comparable, based on the previous study the sampling number is set to 1,000. In this way, each raw data sample of emission data possesses 1,000 simulation-constructed randomly selected meteorological conditions, which are estimated by combining 1,000 meteorological terms (resample’s) with the same emission term (fixed) using the trained Random Forest model, and the estimation yields 1,000 PM_2.5_ concentrations under the same emission but different meteorological conditions, which are averaged to obtain the 1,000 PM_2.5_ concentrations were averaged to finally obtain the PM_2.5_ concentration formed under the conditions of the average meteorological field for that emission term (time term). The above operation was repeated for each piece of raw data to obtain the PM_2.5_ concentration under the condition of average meteorological field for each emission item (time item). It is crucial to clarify that this ‘emission signal’ represents the manifestation of emissions as pollution concentrations under average meteorological conditions (PM_dw), which fundamentally differs from the direct emission quantities (e.g., in tons per year) reported in an emission inventory. While our deweathering approach provides a more stable spatiotemporal pattern reflective of underlying emission trends by removing meteorological variability, it does not directly quantify absolute emission rates from specific sources nor can it delineate individual emission sources. Therefore, direct comparisons with emission inventories, which capture detailed source-specific emission quantities, should be made with an understanding of these distinct methodological objectives.

## 3 Results

### 3.1 Model accuracy evaluation

**Figure 2:**
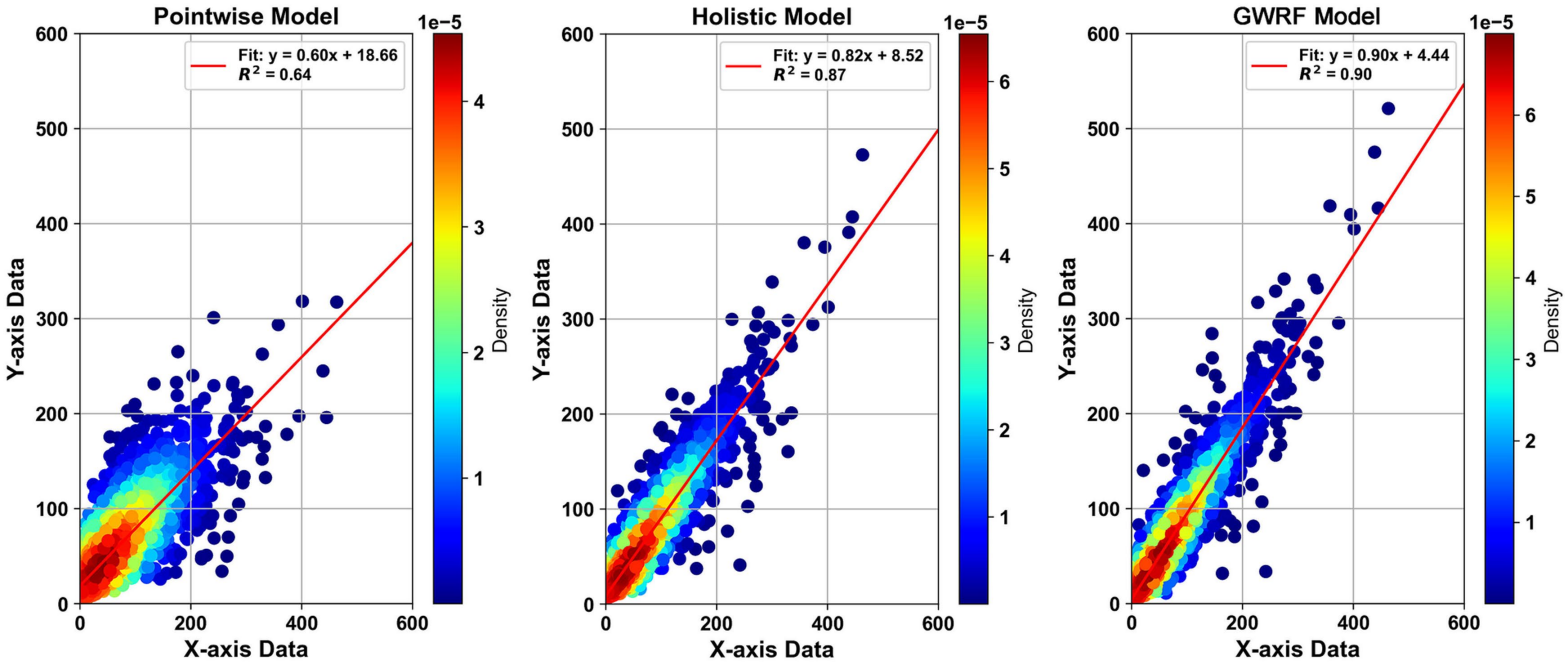
Model Performance and GWRF model Analysis. Panels show probability density scatter plots comparing actual data (*X*-axis) to model predictions (*Y*-axis), with color gradients representing data point density. Specifically, Pointwise, Holistic, and GWRF model predictions are illustrated, highlighting how closely each model’s predictions align with actual observations.

This study applied an optimized random forest model to estimate PM_2.5_ levels across the country, achieving high estimation accuracy. The GWRF model exemplifies exceptional performance in predicting PM_2.5_ concentrations, demonstrating superior accuracy not only through traditional metrics such as RMSE, MAPE, and MAE but also in spatial effectiveness across China’s diverse geographic landscape. In Table 2 and Figure 2: the GWRF model achieves the lowest RMSE at 12.51 μg/m^3^ and excels in MAPE and MAE with scores of 17.73% and 6.88 μg/m^3^, respectively, outperforming the Pointwise and Holistic models. Combining these insights, the GWRF model not only stands out for its high precision and adaptability but also for its utility in enhancing deweathering processes and regional air quality assessments, making it a prime tool for environmental policy and health risk evaluations across varied geographic settings. The model demonstrates excellent estimation performance and can be applied to the next Deweather stage.

**Table 2:** Model accuracy comparison.

Model	*R* ^2^	RMSE	MAPE	MAE	*R*
GWRF	0.90	12.51	17.73	6.88	0.95
RF holistic model	0.87	15.62	20.91	8.28	0.89
RF pointwise model	0.64	18.32	31.80	12.32	0.82

### 3.2 Spatial and temporal characteristics of economic development and air pollution in China

**Figure 3:**
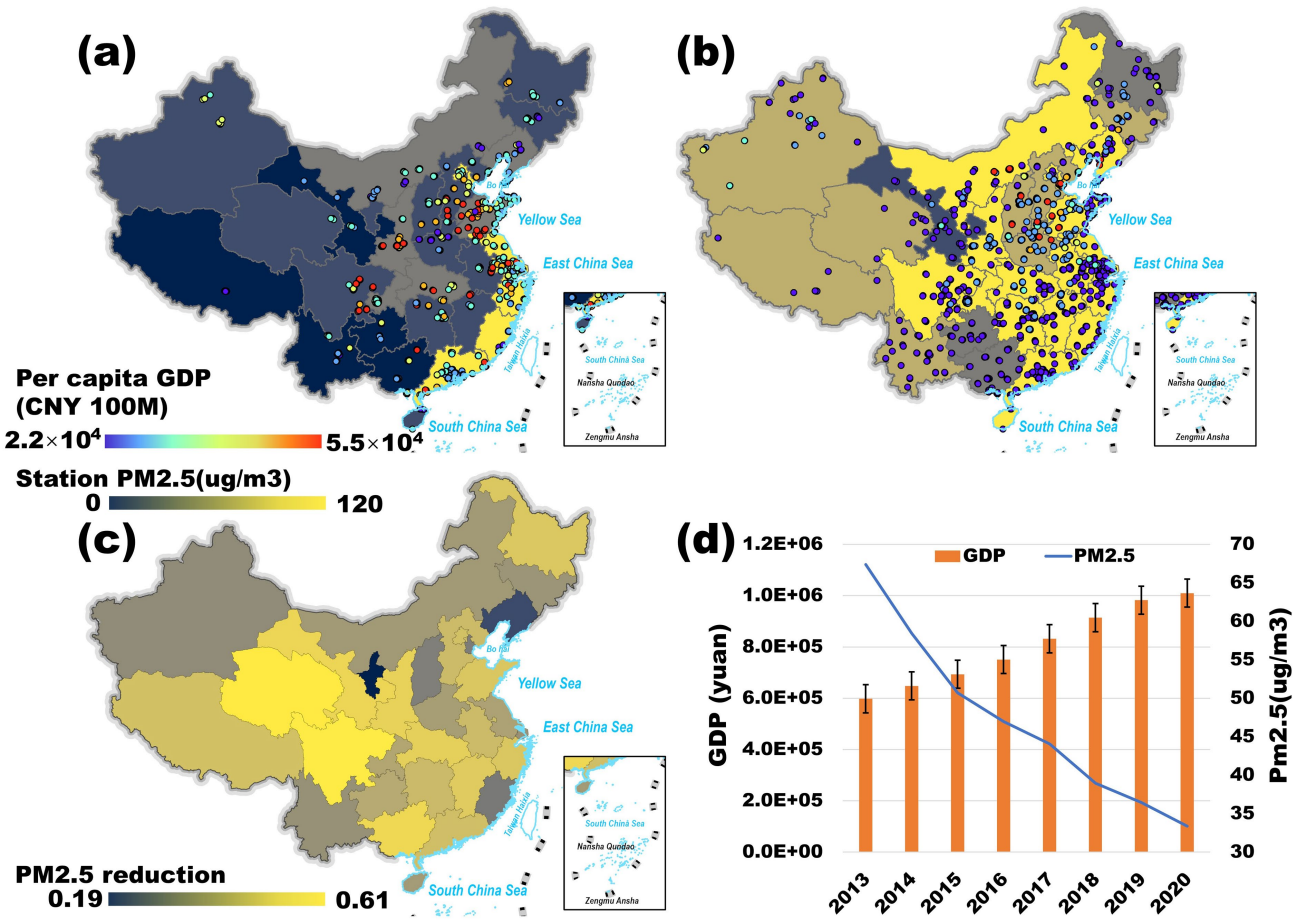
**(a,b)** Spatial distribution of GDP per capita (Province Shading) and PM_2.5_ (Hotspot Markers) in 2013 and 2020; **(c)** Decrease in PM_2.5_ (%) in each province from 2013 to 2020; **(d)** Time series of changes in the national total GDP and PM_2.5_ mean values from 2013 to 2020. Adapted with permission from “(a,b) Spatial distribution of PM2.5 in 2013 and 2020; **(c)** decrease in PM2.5 (%) in each province from 2013 to 2020; (d) time series of changes in the national total GDP and PM2.5 mean values from 2013 to 2020” by Wu et al. (58), licensed under CC BY 4.0.

Figure 3 shows the changes in the temporal and spatial distribution of PM_2.5_ and GDP in China. From a temporal perspective, China’s overall PM_2.5_ emissions have been steadily declining since 2013, with regional variations in the rate of reduction. The most significant declines were observed in highly polluted regions such as North, Central, and Western China, while the decrease was less pronounced in Northwest China.

Spatially, PM_2.5_ concentrations exhibit distinct regional patterns. First, eastern and central China experience higher PM_2.5_ levels than the western region, with developed regions exhibiting greater emissions than less developed areas. This pattern aligns with the spatial concentration of GDP per capita in China’s three major urban agglomerations—Beijing-Tianjin-Hebei, the Yangtze River Delta, and the Pearl River Delta—where population density, industrial activity, and economic output are highest (35, 36). Second, a pronounced north–south disparity exists, with PM_2.5_ concentrations significantly higher in the north than in the south, primarily due to centralized heating and climatic conditions along the Qinling-Huaihe dividing line (37, 38). Third, population density strongly correlates with pollution severity, as densely populated areas tend to experience higher PM_2.5_ levels. Finally, coastal regions generally exhibit lower PM_2.5_ concentrations than inland areas, largely due to more favorable meteorological conditions and atmospheric dispersion processes.

The relationship between GDP and PM_2.5_ concentrations does not exhibit a straightforward correlation, which can be attributed to several factors. First, geographic conditions play a key role. The Beijing-Tianjin-Hebei, Yangtze River Delta, and Pearl River Delta economic zones are located along the coast, benefiting from maritime transportation and trade. Additionally, regional meteorological conditions and pollutant dispersion influence population exposure levels, further shaping PM_2.5_ distribution patterns. Second, national policies promoting industrial upgrading and clean technology adoption have led to a continuous reduction in industrial emissions, particularly in economically developed regions where pollution control measures have become more stringent, aligning with national air quality standards.

As shown in Figure 3d, since 2013, PM_2.5_ concentrations have exhibited a clear downward trend, while China’s GDP has steadily increased, reflecting the country’s commitment to sustainable environmental development alongside economic growth. Figure 3c further illustrates the percentage reduction in PM_2.5_ across provinces from 2013 to 2020. Notably, pollution reductions were more pronounced in economically less developed regions such as Southwest and Northwest China, whereas reductions in economically developed regions were relatively modest.

**Figure 4:**
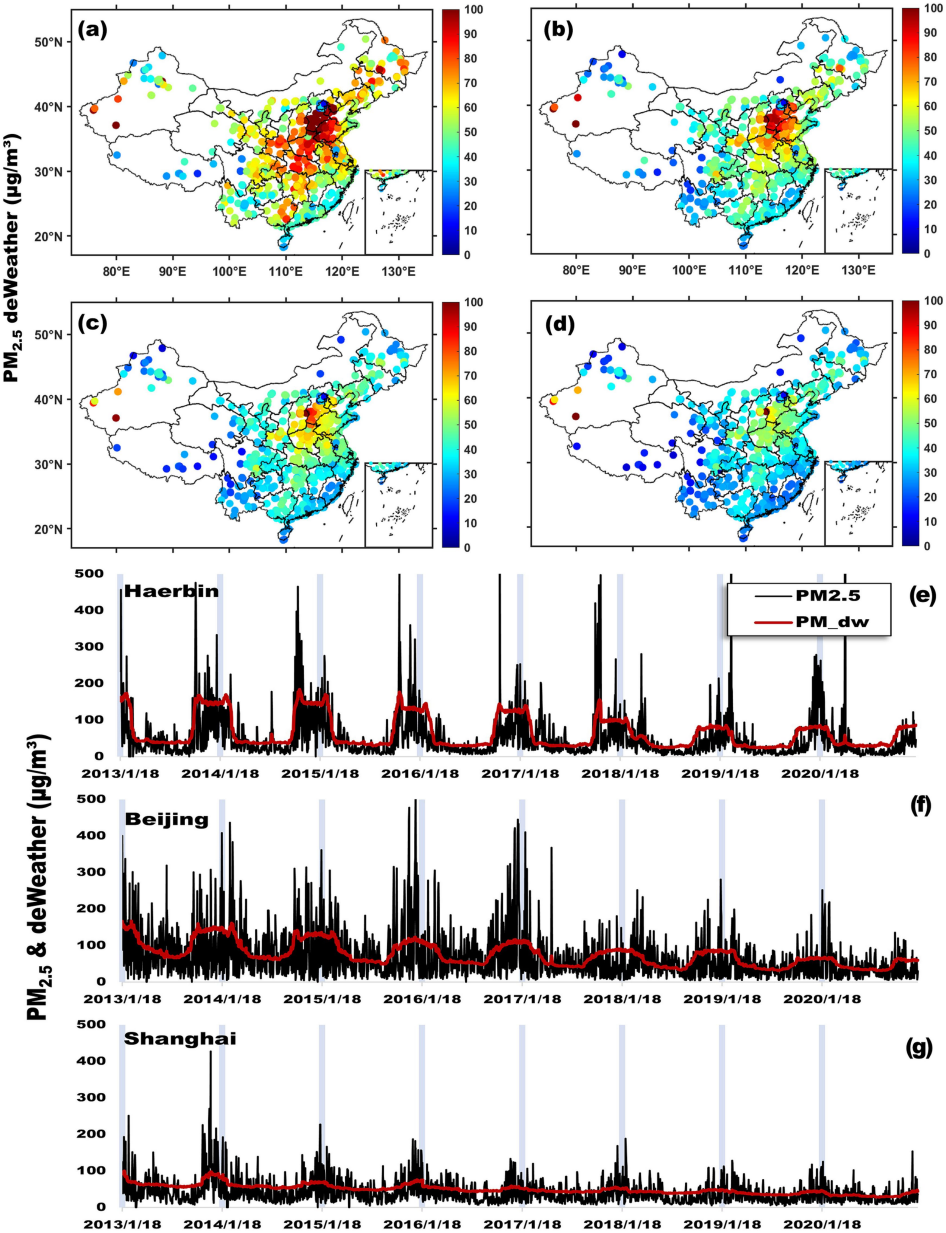
Deweathered and actual PM_2.5_ concentrations across major Chinese urban clusters. Panels **(a–d)** depict the spatial distribution of deweathered PM_2.5_ (PM_dw) concentrations across China for the years 2014, 2016, 2018, and 2020, respectively, with varying colors indicating different concentration levels. Panels **(e–g)** focus on time series analysis of both actual measured (black line) and deweathered (red line) PM_2.5_ concentrations in Harbin, Beijing, and Shanghai, representing the Eastern Three Provinces, Jing-Jin-Ji, and Yangtze River Delta urban clusters. These panels illustrate daily fluctuations and broader pollution trends from 2013 to 2020, highlighting the environmental challenges and seasonal variations faced by these key metropolitan areas.

The deweathering method produced the final PM_dw, enabling a more precise analysis of pollution trends. Figure 4 illustrates the temporal and spatial variations of PM_2.5_ concentrations across major urban clusters in China from 2013 to 2020, comparing deweathered and actual data. The findings reveal critical insights into pollution trends: Temporal Variations: PM_2.5_ levels in cities such as Harbin, Beijing, and Shanghai have shown a declining trend over the years, though with seasonal fluctuations. The time-series data (Figures 4e,f,g) indicate that both actual and deweathered PM_2.5_ levels exhibit seasonal peaks, particularly in winter, due to increased heating demand and stagnant atmospheric conditions that trap pollutants. The deweathered data (red lines) present a smoother trend with reduced peaks, suggesting that meteorological influences exacerbate seasonal pollution spikes. Removing these effects provides a clearer assessment of emission-driven pollution trends. Spatial Variations: The maps in panels Figure 4a through Figure 4d illustrate the evolution of PM_2.5_ concentrations in selected years. From 2014 to 2020, air quality has generally improved, particularly in major cities, largely due to stringent pollution control measures. However, significant regional disparities persist, with northern China consistently exhibiting higher pollution levels than the south. This pattern reflects ongoing challenges related to industrial emissions and coal dependency in northern regions. The comparison between actual and deweathered PM_2.5_ data highlights the substantial impact of meteorological conditions on perceived pollution levels. While policy interventions have contributed to pollution reductions, their effectiveness varies significantly across regions, and weather effects often obscure actual trends in raw data analyses.

Between 2013 and 2020, China’s economy maintained steady growth while pollution control measures led to notable improvements. However, regional economic disparities have resulted in significant differences in pollution exposure. As urbanization accelerates and green development policies advance, understanding the extent to which emission reductions contribute to inter-regional environmental equity becomes increasingly important. This necessitates a closer examination of how different regions have benefited from emission control policies and whether disparities in pollution exposure have narrowed over time.

### 3.3 Regional equity analysis of emission reductions

**Figure 5:**
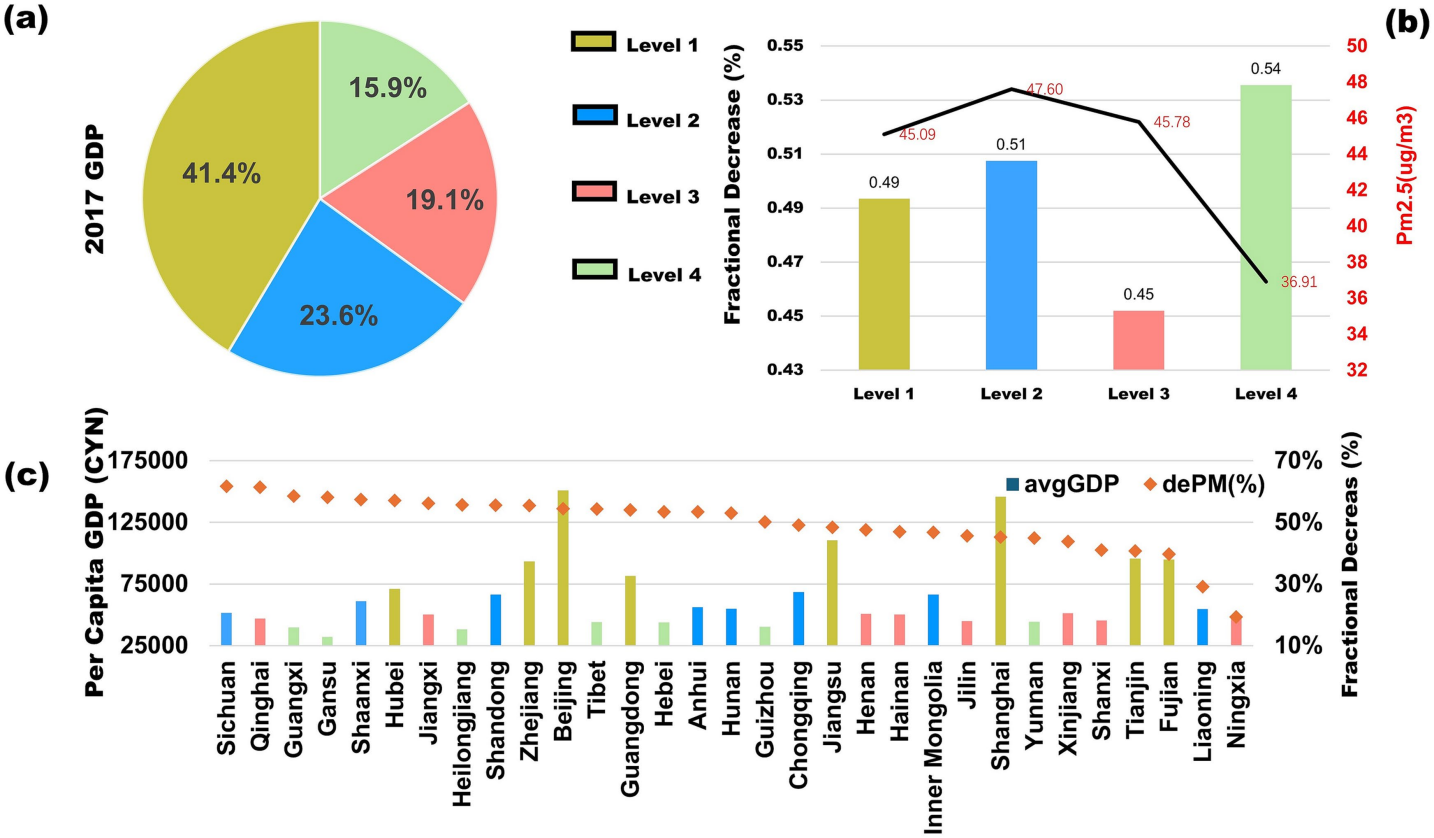
Four subgroups of Chinese provinces based on GDP, **(a)** share of GDP contribution of each subgroup in 2017 **(b)** PM_2.5_ Reduction (2013–2020) and Average PM_2.5_ Concentration: The colored bars represent the fractional decrease in PM_2.5_ from 2013 to 2020, while the yellow crosses indicate the average PM_2.5_ concentration during the same period. **(c)** *Per Capita* GDP and PM_2.5_ Reduction (2013–2020) by Province: The colored bars represent per capita GDP in 2017 for the four subgroups, while the black line shows the fractional decrease in PM_2.5_ concentration from 2013 to 2020 for each province. Adapted with permission from “Four subgroups of Chinese provinces based on GDP” by Wu et al. (58), licensed under CC BY 4.0.

This study categorizes China’s 32 provinces into four groups based on GDP per capita (Figure 5): L1 (high-income group) includes provinces in the top 25% of GDP per capita, primarily located along the eastern coast, such as Beijing and Shanghai, where industrialization is highly advanced (39). L2 (middle-high-income group) consists of provinces ranking between 25 and 50%, mostly in central and western China, balancing industrial and agricultural output, such as Chongqing and Anhui (40). L3 (low-middle-income group) includes provinces in the 50–75% GDP per capita range, widely distributed across inland regions (40). L4 (low-income group) represents provinces in the bottom 25%, mainly in remote areas relying on tourism and low-end agriculture, such as Guangxi and Gansu (41).

From Figure 5a, L1 contributed the highest share of GDP in 2017 (41.4%), while L2 and L3 accounted for around 20% each, and L4 had the smallest share at 15.9%. Figure 5b shows that the average PM_2.5_ concentration in L4 was 37 μg/m^3^, with a reduction rate of 54% from 2013 to 2020. In contrast, L1, L2, and L3, which have higher economic levels and greater pollution burdens, exhibited PM_2.5_ levels between 45 and 47.6 μg/m^3^, with a reduction rate of only 45–50%. As illustrated in Figure 5c, the L1 group ranks at a mid-to-low level in national emission reductions, whereas L3 and L4 show significantly higher percentage reductions, suggesting that low-pollution, economically disadvantaged regions bear a disproportionately larger burden of emission reduction efforts.

The industrial structure and energy consumption patterns of less-developed regions are relatively homogeneous and easier to adjust, making short-term emission reduction policies highly effective. However, in the long term, these measures increase marginal abatement costs and economic pressures, potentially constraining regional economic growth (42). Moreover, disproportionate emission reduction mandates and pre-existing regional economic disparities may further exacerbate interregional inequality (42, 43). This reflects the interplay between regional resource allocation, policy efficiency, and the economic trade-offs associated with China’s ongoing environmental governance.

### 3.4 Differences in pollution exposure between urban and rural areas

To further examine disparities in PM_2.5_ exposure across different social groups, while excluding meteorological influences, this study investigates pollution exposure patterns in the context of urban–rural differences and population migration.

**Figure 6:**
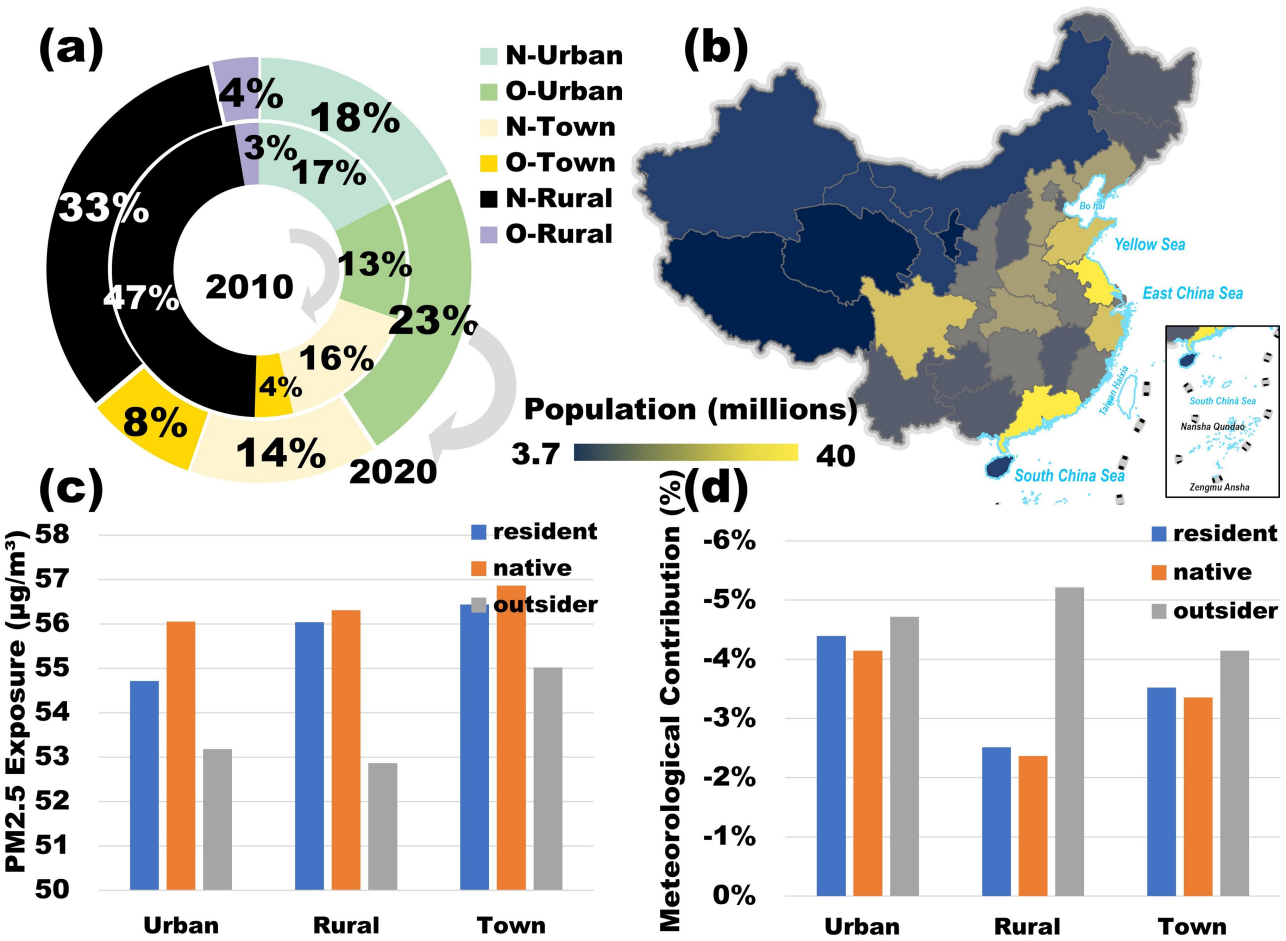
**(a)** National 2010 and 2020 N-Urban, O-Urban, N-Town, O-Town, N-Rural, and O-Rural population share, where O represents outsiders and N represents natives. **(b)** National provincial city population distribution in 2020. **(c)** Urban, town, and rural populations’ average PM_dw exposure for residents, natives, and outsiders from 2013 to 2020. **(d)** Meteorological contribution to average PM_dw exposure for residents, natives, and outsiders in urban, town, and rural populations from 2013 to 2020. Adapted with permission from “Spatial distributions of county-level populations in 2020” by Wu et al. (58), licensed under CC BY 4.0.

Figure 6a illustrates changes in China’s urban and rural population distribution, as well as local and foreign residents, between 2010 and 2020 (6th and 7th Census). The urban population share increased from 30% (N: 17%, O: 13%) to 41% (N: 18%, O: 23%), with most growth driven by an influx of foreign residents. Meanwhile, town populations grew from 20% (N: 16%, O: 4%) to 22% (N: 14%, O: 8%), again largely due to an increase in foreign residents. In contrast, the rural population declined significantly from 50% (N: 47%, O: 3%) to 37% (N: 33%, O: 4%), with most of the loss attributed to local migration. These patterns underscore that rural-to-urban migration has been a dominant feature of China’s urbanization process in recent years.

Figure 6b presents China’s 2020 population distribution, revealing a high-density population in the east and a sparse population in the west. Urban clusters such as the Yangtze River Delta, Pearl River Delta, and Sichuan Basin have become key industrial hubs, attracting large numbers of migrant workers through advanced manufacturing, services, and high-tech industries (44). However, industrial expansion, coupled with population concentration, has intensified pollution exposure, as the convergence of industrial pollution and high population density amplifies environmental risks (45, 46). This interplay between urbanization, economic growth, and pollution dynamics highlights the urgent need for balanced development strategies to mitigate environmental inequalities.

Figure 6c illustrates the average PM_dw exposure levels among urban, rural, and township populations, as well as local and migrant residents, from 2013 to 2020. The results indicate a hierarchical pollution exposure pattern, where towns experience the highest exposure, followed by townships, and then cities. Additionally, local populations face higher pollution exposure than migrant populations. This trend can be attributed to urban industrial advantages, which attract large numbers of migrants from rural and township areas, fostering labor and market expansion that accelerates industrial upgrading and stricter environmental regulations (47). Consequently, high-polluting industries are relocated to peripheral towns (45), leveraging lower land and energy costs as well as convenient transportation networks, while reducing direct environmental impacts on densely populated urban centers. As a result, cities and towns bear the highest burden of air pollution exposure.

Figure 6d presents the meteorological contribution to PM_dw exposure across different population categories. The results suggest that meteorology generally plays a pollution-dissipating role, with wind and relative humidity being the dominant factors in pollutant transport, chemical reactions, and deposition (48). Cities and towns benefit the most from meteorological dispersion effects, with migrant populations experiencing greater reductions in exposure than local residents. This may be due to the higher pollution base and population density in urban areas, which, combined with the urban heat island effect, enhances localized wind fields and convection currents, creating favorable meteorological conditions for pollutant dispersion and deposition.

### 3.5 Differences in PM_2.5_ exposure between social groups

**Figure 7:**
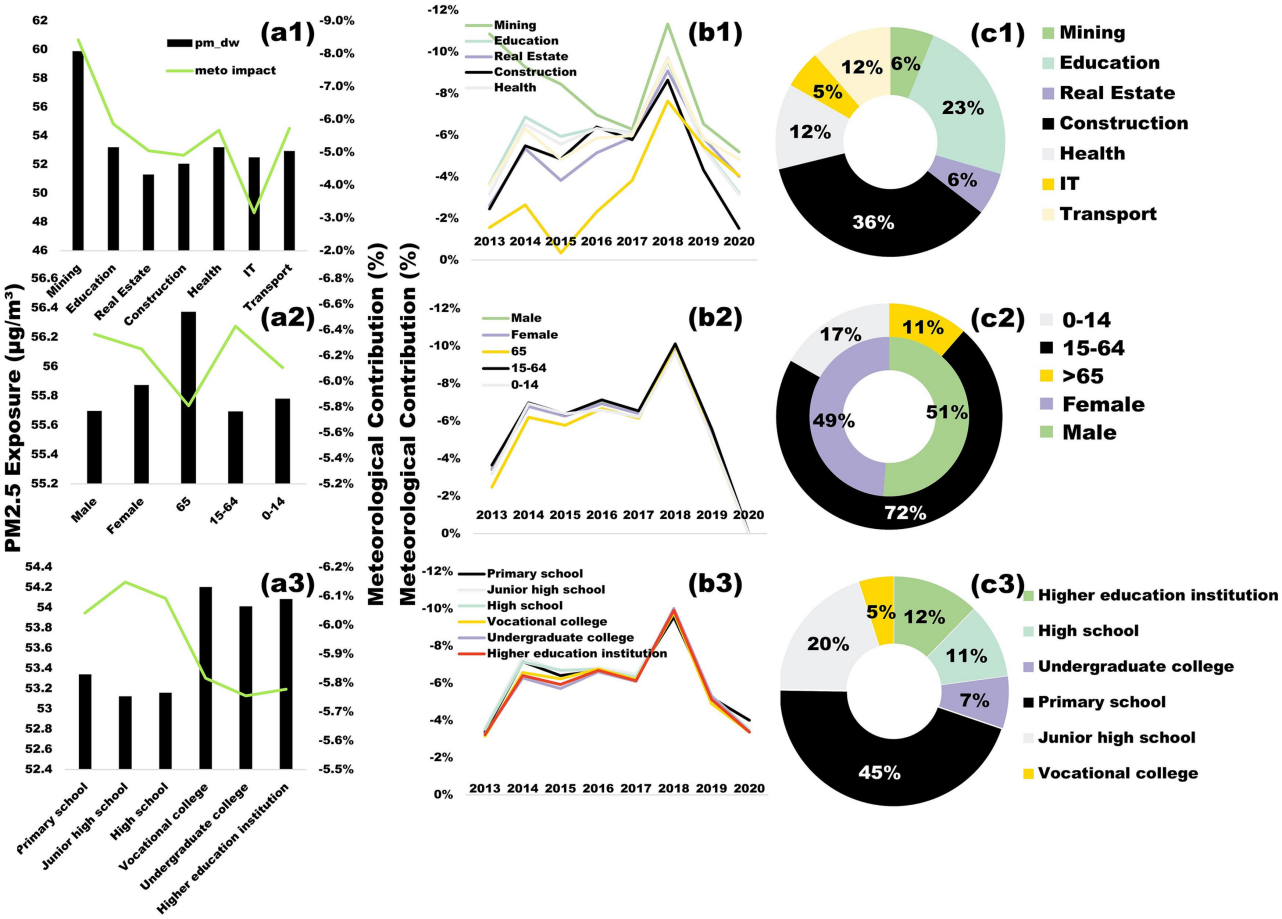
**(a1–a3)** Differences in PM_dw exposure among populations with different occupations, age groups, genders, and education levels, averaged over 2013–2020, the green dashed line represents the meteorological contribution. **(b1–b3)** Effect of average weather on PM_dw exposure among populations with different occupations, age groups, genders, and education levels, averaged over 2013–2020. **(c1–c3)** Percentage of populations with different occupations, age groups, genders, and education levels, 2017. Adapted with permission from “(a,c,e) Differences in average PM2.5 exposure among populations with different occupations, age groups, genders, and education levels in 2020 (the red line indicates the percentage decrease in 2020 compared to 2013). (b,d,f) Proportions of populations with different occupations, age groups, genders, and education levels in 2017” by Wu et al. (58), licensed under CC BY 4.0.

Analysis of population-weighted PM_2.5_ exposure (PM_dw) across industries reveals notable disparities (Figure 7a1). Mining workers experience the highest PM_dw levels (59.89 μg/m^3^), primarily due to prolonged exposure to coal-related pollution during extraction, transport, and combustion (49–51). This pattern aligns with previous findings linking northern China’s coal-dependent heating systems to elevated emissions (52–55). Meteorological dispersion has the strongest impact in mining (−0.084), indicating that weather conditions significantly mitigate pollution exposure in this sector. In contrast, IT workers experience lower PM_dw levels (52.50 μg/m^3^) with minimal meteorological influence (−0.032), likely due to their predominantly indoor work environments. Construction workers, despite comprising 36% of the workforce, exhibit moderate PM_dw levels (53.21 μg/m^3^), reflecting the combined effects of outdoor exposure and favorable dispersion conditions.

Gender and age differences further shape PM_2.5_ exposure patterns (Figure 7a2). Females exhibit slightly higher PM_dw levels (55.87 μg/m^3^) than males (55.69 μg/m^3^), despite males being overrepresented in high-exposure industries such as construction and transport. This discrepancy may result from females’ greater involvement in household cooking and their higher likelihood of residing in pollution-prone rural or peri-urban areas. Meteorological dissipation effects favor males slightly more (−6.3% vs. −6.2% for females), possibly due to males’ increased outdoor activity in well-ventilated environments.

Age-stratified data indicate that older populations (65 + years) face the highest PM_dw levels (56.37 μg/m^3^), exceeding those of younger age groups (55.78 μg/m^3^ for 0–14 years). This trend is consistent with their higher concentration in regions characterized by aging infrastructure, coal-based heating, and limited green spaces (56). Conversely, younger populations benefit from pollution control policies in regulated school zones, which help reduce their overall exposure risks.

Educational background also correlates with PM_dw exposure (Figure 7a3). Individuals with vocational (54.20 μg/m^3^) and undergraduate education (54.01 μg/m^3^) face higher exposure than primary school cohorts (53.34 μg/m^3^), reflecting their occupational concentration in pollution-intensive industrial hubs. Weaker meteorological dispersion effects (−5.78% for higher education vs. −6.04% for primary education) further exacerbate exposure in these regions. In contrast, lower-educated populations, less engaged in formal industries, have lower pollution exposure but remain socioeconomically vulnerable, highlighting the need for further investigation.

Figure 7b presents the time-series variation of meteorological effects on PM_2.5_ exposure. The results indicate that meteorological conditions generally mitigate pollution exposure, following a rise-and-fall trend from 2013 to 2020, peaking in 2018. This peak may be attributed to temporary improvements in meteorological conditions such as increased wind speed and precipitation, which enhanced pollutant dispersion and deposition. However, recent shifts suggest a reversal, with meteorological conditions now favoring pollution retention.

These findings highlight the complex interplay between industrial policy, demographic dynamics, and environmental governance in shaping PM_2.5_ exposure disparities. Mitigation strategies should be tailored to sector-specific risks, with a particular focus on protecting vulnerable populations in high-pollution regions through enhanced environmental policies and industrial regulation. Addressing these inequities requires comprehensive pollution control measures, targeted emission reduction strategies, and sustainable urban planning to minimize exposure risks across different socio-economic groups.

To deeply investigate the driving factors of PM_2.5_ exposure inequality and its manifestation under meteorological and emission influences, this study conducted a comparative analysis of the degree and sources of inequality in exposure to raw PM_2.5_ (PM) and deweathered PM_2.5_ (PM_dw). We employed the Lorenz curve, Gini coefficient, and Theil contribution decomposition method. For analyses concerning occupational and education level groups, we examined data for 2013 and 2019. For analyses concerning urban–rural and local/migrant groups, we examined data for 2010 and 2020.

**Figure 8:**
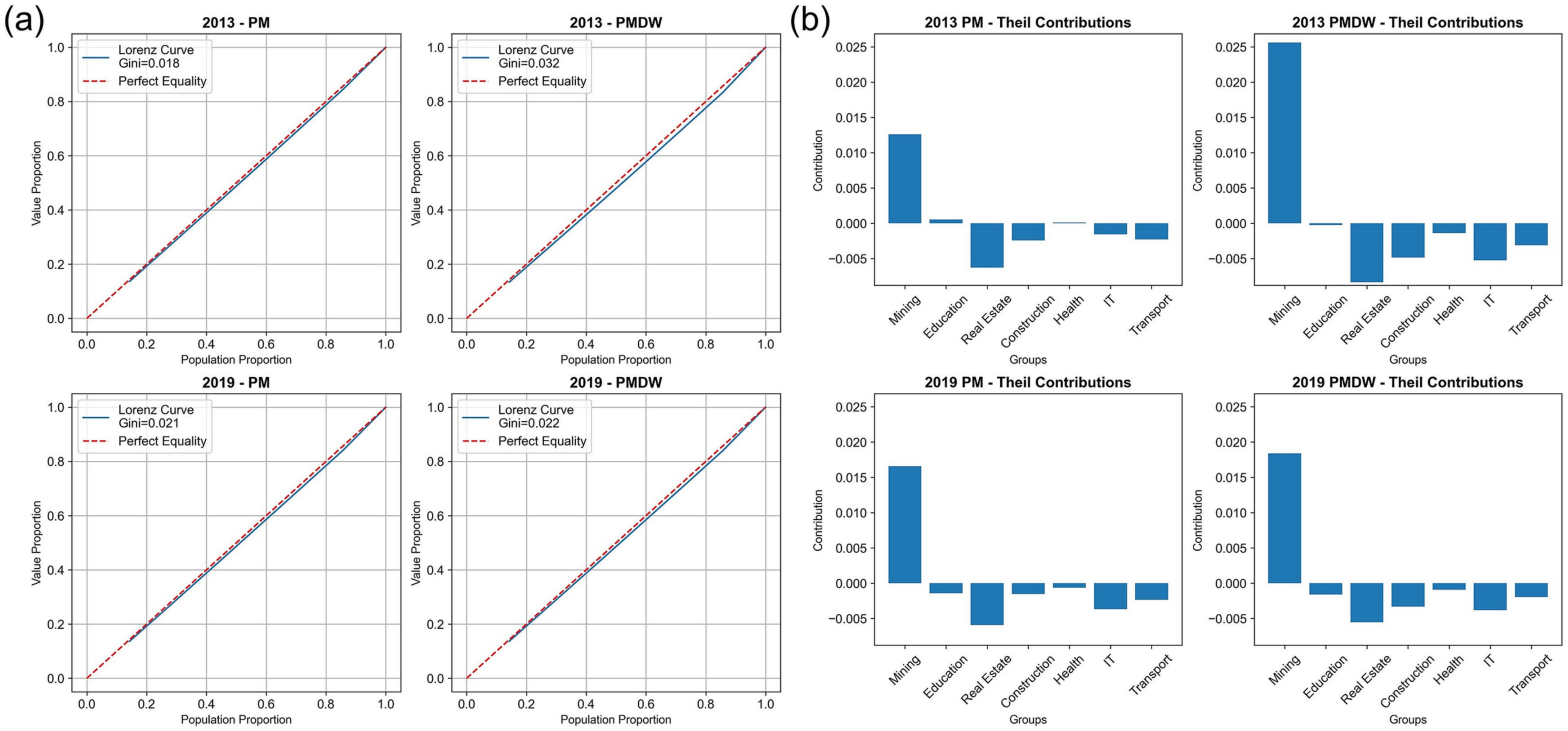
Comparison of actual (PM) and deweathered (PM_dw) PM_2.5_ exposure inequality among occupational groups in 2013 and 2019. **(a)** Lorenz curves and Gini coefficients. **(b)** Theil contributions by occupational group.

Figure 8 illustrates the inequality characteristics of PM and PM_dw exposure among different occupational groups. From the Lorenz curve and Gini coefficient (Figure 8a), in 2013, the Gini coefficient for raw PM was 0.018, while for PM_dw, it was 0.032. This difference indicates that meteorological conditions played a role in mitigating inequality in occupational exposure. However, by 2019, the Gini coefficient for raw PM decreased to 0.021, and for PM_dw, it was 0.022. The mitigating effect of meteorology drastically decreased from 43.7% in 2013 to 4.5% in 2019, indicating a significant reduction in meteorology’s role in alleviating inequality. The Theil contribution analysis (Figure 8b) further reveals that the mining industry, within occupational groups, is a population segment that exacerbates inequality, and is significantly higher than other groups.

Supplementary Figure S1 focuses on the inequality performance of PM and PM_dw exposure among different education level groups. Changes in the Gini coefficient indicate that meteorology presents a slight exacerbating effect on exposure inequality within the education level dimension. The Theil contribution analysis further shows that the higher the education level, the more pronounced the inequality. Supplementary Figure S2 reveals that the burden of PM_2.5_ exposure inequality was more significantly borne by local populations. Specifically, in 2010, rural populations experienced a greater degree of pollution inequality. The meteorological effect exacerbated urban–rural exposure inequality in 2010, contributing 43.7%; however, by 2020, this influence significantly improved, with meteorology shifting to alleviate pollution inequality in town areas. This change highlights the complex influence of meteorological conditions on PM_2.5_ exposure inequality across different years and socio-economic dimensions.

## 4 Discussion

This study comprehensively analyzed population PM_2.5_ exposure in China from 2013 to 2020, uniquely disentangling the complex interplay of emissions, meteorology, and socioeconomic disparities. Leveraging a robust GWRF model, which demonstrated superior performance (RMSE of 12.51 μg/m^3^, MAPE of 17.73%, MAE of 6.88 μg/m^3^) over traditional models, we assessed population-weighted exposure across 1,317 county towns.

Our findings reveal a nuanced picture of environmental inequality. Lower-income regions (L4) achieved the highest PM_2.5_ reduction (54%), while wealthier regions (L1–L3) saw lower reductions (45–50%), highlighting a disproportionate emission reduction burden. Despite overall improvements, residents in less-developed areas continue to face higher exposure, and vulnerable populations—including coal miners (enduring the highest PM_dw levels at 59.89 μg/m^3^), the educated, women, and the older adult—disproportionately suffer from elevated exposure risks.

Crucially, our study illuminates the complex and evolving role of meteorological conditions in modulating exposure inequality. While meteorology generally mitigates PM_2.5_ exposure, with the most significant dispersion effect in 2018, its specific impact on inequality varies dynamically. Notably, meteorology’s role in mitigating inequality in occupational exposure significantly decreased from 43.7% in 2013 to 4.5% in 2019. Concurrently, its exacerbating effect on urban–rural inequality, contributing 43.7% in 2010, drastically reduced by 2020, even shifting to a slight alleviating role. The enhanced stability of PM_dw compared to raw PM_2.5_ provides a clearer signal for understanding underlying emission trends, critical for policy evaluation.

In conclusion, achieving equitable environmental governance and robust pollution control in China necessitates multifaceted policy approaches. Policies must not only address regional economic disparities and prioritize protection for disadvantaged communities through targeted interventions (e.g., sector-specific controls, clean energy transitions, infrastructure upgrades) but also account for the complex and evolving modulating role of meteorological conditions on exposure inequality. This comprehensive understanding is vital for developing effective strategies that promote both environmental sustainability and social justice.

## Data Availability

The datasets presented in this study can be found in online repositories. The names of the repository/repositories and accession number(s) can be found below: https://zenodo.org/records/14879617.

## Footnotes

^1^ https://data.stats.gov.cn
