# Supplementary material for: Decoding population PM2.5 exposure in China: interplay of emissions, meteorology, and inequality (2013–2020)
Source: Front Public Health. 2025 Aug 11;13:1577897. doi: 10.3389/fpubh.2025.1577897 (PMC12376197; doi:10.3389/fpubh.2025.1577897)
Supplement: Supplementary file 1 [file Data_Sheet_1.docx]

Supplementary Material

# Supplementary Figures and Tables

## Supplementary Figures
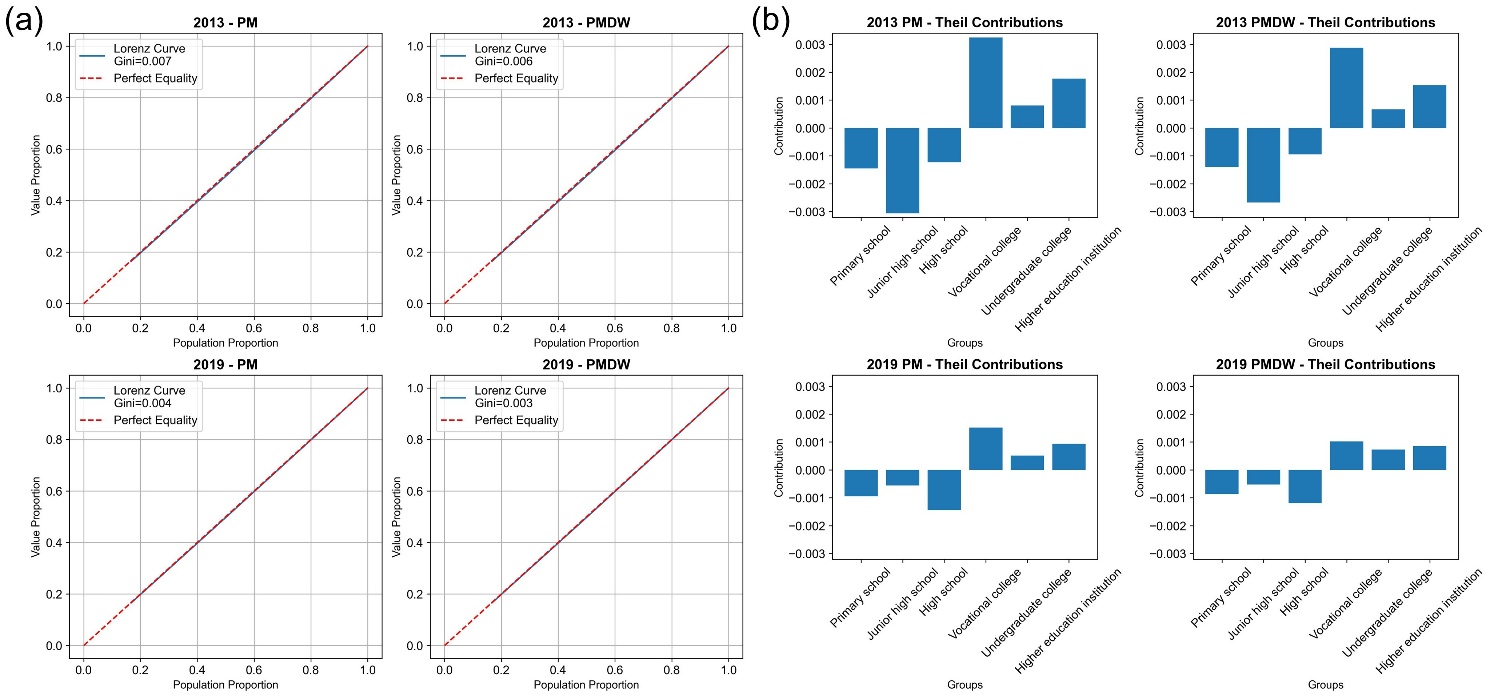
Supplementary Figure 1. Analysis of PM2.5 Exposure Inequality Across Education Level Groups (2013, 2019). (a) Lorenz Curves and Gini Coefficients. (b) Theil Contributions by Education Level Group.
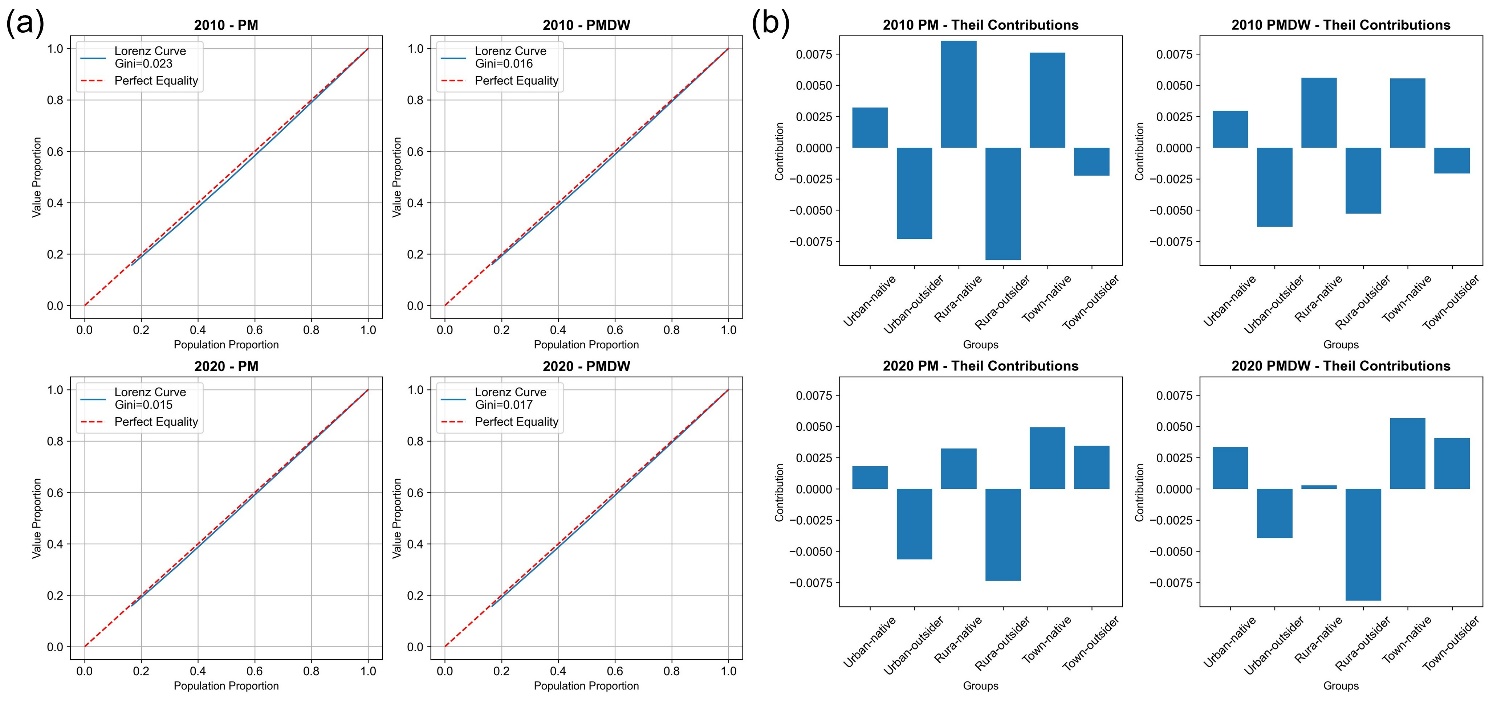
Supplementary Figure 2. Analysis of PM2.5 Exposure Inequality Across Urban-Rural and Local/Migrant Groups (2010, 2020). (a) Lorenz Curves and Gini Coefficients. (b) Theil Contributions by Urban-Rural and Local/Migrant Group.
